# Degradation of Losartan Potassium Highlighted by Correlated Studies of Photoluminescence, Infrared Absorption Spectroscopy and Dielectric Spectroscopy

**DOI:** 10.3390/pharmaceutics14112419

**Published:** 2022-11-09

**Authors:** Mirela Paraschiv, Ion Smaranda, Irina Zgura, Paul Ganea, Madalina Chivu, Bogdan Chiricuta, Mihaela Baibarac

**Affiliations:** 1 National Institute of Materials Physics, Atomistilor street 405A, P.O. Box MG-7, 077125 Bucharest, Romania; 2 Faculty of Physics, University of Bucharest, P.O. Box MG-11, 077125 Bucharest, Romania; 3 SC Apel Laser SRL, 25 Vanatorilor St, 077135 Mogosoaia, Romania

**Keywords:** losartan potassium, photodegradation, photoluminescence, IR spectroscopy, dielectric spectroscopy

## Abstract

In this paper, new results on the degradation of losartan potassium (LP, (**1**)), in the absence and presence of excipients, which was induced by UV light, the acid character of phosphate buffer solution (PBS) and alkaline medium, respectively, are reported through correlated studies of FTIR spectroscopy, photoluminescence and dielectric spectroscopy. The photoluminescence (PL) spectra of LP and the drug marked under the name Lorista (LO) are characterized by intense emission bands, peaking at 378 nm and 380 nm, respectively, accompanied by low intensity bands with a maximum at ~450–460 nm. Photodegradation of LO in a solid state is evidenced by a decrease in the intensity of the PL band at 380 nm, a variation that originates both in the adsorption of water vapors from the air and in the interaction of LP with excipients such as cornstarch, silicon dioxide and cellulose. The LP-water interaction is described, taking into account the main electrical parameters, i.e., complex dielectric permittivity and electrical conductivity. Photodegradation of LP and LO also induces an increase in the intensity of the emission band, at ~450–460 nm. The influence of acid and alkaline medium on the LO degradation is analyzed using phosphate buffer (PBS) and NaOH solutions, respectively. In both cases, a decrease in the intensity of the PL band, at 380 nm, is reported. The intensity diminution of the PL spectra of NaOH-reacted LP and LO is the result of the formation of the photodegradation product N-methanolamide-{[2′-(1H-tetrazol-5-yl)(1,1′-biphenyl)-4-yl]methyl} (**2**). This compound was proven by the studies of FTIR spectroscopy achieved on LP and NaOH-reacted LP. The appearance of the IR band at 1740 cm^−1^ and the increase in the absorbance in the IR band at 1423 cm^−1^ indicate that the photodegradation product (**2**) contains the C=O and C-OH functional groups.

## 1. Introduction

Losartan potassium (LP), know under the name of 2-butyl-4-chloro-1-{[2′-(1H-tetrazol-5-yl)(1,1′-biphenyl)-4-yl]methyl}-1H-imidazole-5-methanol monopotassium salt (**1**), is used as an active substance in the pharmaceutical product marked under the name Lorista (LO) and is administered for high blood pressure [1], kidney disease in diabetic persons [s2] and heart failure [2]. Despite its therapeutic effect, some side effects are reported [2,3]. Various methods for LP (**1**) detection have been adopted, for instance: high-performance thin-layer chromatography [4], fluorescence densitometry [5], fluorometric detection [6], mass spectrometry [7], UV-VIS spectroscopy [8,9] and cyclic voltammetry [10]. The analysis of LP by UV-VIS spectroscopy has also been used to remove the contaminants under UVC light, when the photolysis processes of LP (**1**), induced by H_2_O_2_ and Na_2_S_2_O_8_, were reported [11]. Both the detection and removal methods of LP (**1**) involve, in the first stage, a dissolution of LP (**1**) in aqueous solutions that have different pH values. 

Compared to these studies, in this manuscript attention will be given to: i) monitoring the LO degradation, caused by the UV light, the pH of phosphate buffer solution (PBS) and alkaline medium, respectively, through photoluminescence (PL) and infrared (IR) spectroscopy; ii) the contribution of the excipients on the LP photodegradation; and iii) the understanding of the influence of the water vapors from air on the LO photodegradation. These studies will allow us: (i) to anticipate the handling or storage conditions of LO in the presence of air and UV light during the therapeutic treatment, as well as of the pH conditions used for the detection of LP (**1**) in the various pharmaceutical formulations when methods such as liquid chromatography, UV-VIS spectroscopy, photoluminescence or cyclic voltammetry are used; (ii) to understand the contribution of each excipient used in the preparation of LO on the photodegradation processes of LP, which will indicate the necessary measures to be taken to prevent such processes; and (iii) to illustrate the contribution of water vapors from air on the photodegradation process of LO, the result of which will indicate the need to package this drug under vacuum conditions. Currently, whilst the toxicity of LO is known, the toxic effect of all degradation products has not been analyzed in detail. In this context, two recent studies have demonstrated that the degradation of LO in the presence of UV light leads to the photodegradation of the products, for which an acute toxicity was reported [11,12]. Knowing the evolution of the toxicity of administered drugs and degradation products, as well as ways to reduce the toxicity of these compounds, are key parameters for an effective treatment. 

At present, only one article has focused on the vibrational properties of LP (**1**), highlighted by Raman scattering and FTIR spectroscopy [13]. The degradation pathways and the identification of degradation products was achieved by chromatography [12,14,15], UV-VIS spectroscopy [16], liquid chromatography/mass spectrometry (LC/MS) [17,18] and ^13^C and ^1^H NMR [17]. Six photodegradation products were reported, containing the following functional groups: -NH_2_, -NH-C(R)=NH, -NH-CO-, -COO-, -OH and -CO-NH-CO- [17]. The most stable photodegradation products were found to be N-methanolamide-{[2′-(1H-tetrazol-5-yl)(1,1′-biphenyl)-4-yl]methyl} (**2**) and N-((2’-(1H-Tetrazol-5-yl)-[1,1’-biphenyl]-4-yl)methyl)pentanamide (**3**), and had a molecular weight (MW) equal to 309 and 335, respectively [17]. Spectroscopic investigations have detected carcinogenic nitrosamine impurities over the acceptable intake limit. These were reported to be 26.5 ng/day, in several drugs such as Losartan, Irbesartan, Ranitidine, as well as their source, which led to the withdrawal of these drugs from pharmacies [19,20]. The molecular structure of the compounds (**2**) and (**3**) contain: (i) a five-member ring of the type tetrazole; (ii) two benzene rings and (iii) an amide group. In addition, for the (**2**) compound, the molecular structure also contains a hydroxyl group.

Information regarding the molecular structure of drugs is often reported by IR spectroscopy and Raman scattering. In fact, FTIR spectroscopy in ATR geometry is one of the analytical methods often used to control the quality of drugs. Compared to FTIR spectroscopy, which provides information about symmetric vibrational modes, Raman scattering is a spectroscopic method that provides information about asymmetric vibrational modes. The two methods are currently considered to be complementary. Both FTIR spectroscopy and Raman scattering allow the identification of functional groups in the molecular structure of pharmaceutical compounds and, as shown in numerous items, allow us to highlight the changes induced to vibrational modes by the degradation of pharmaceutical compounds, e.g., [21,22].

Beginning in 2009, another technique used in the drug field is dielectric spectroscopy [23]. The dielectric spectroscopy technique can provide useful information for systems that undergo chemical and/or physical changes as a result of an external stimulus. Thus, in our case, dielectric spectroscopy can give useful data regarding the changes induced in the molecular structure by the irradiation process. It is known that dielectric spectroscopy determines the collective dielectric properties of molecular systems. The technique measures the dynamics of both polar molecules and other localized and delocalized charges. The local mobility of the molecules can be influenced by the specific interactions, such as in hydrogen bonds. In a solution containing the drug intended to be investigated, the interactions between the molecules can influence the intra- and inter-hydrogen bonding, which can further act on the local motions of the molecules; these interaction changes are found in the changes in electrical properties, which are measurable by dielectric spectroscopy [23]. 

In this work, using photoluminescence (PL) and infrared (IR) spectroscopy, new information regarding the effects of UV light, water vapors from air, PBS pH and the alkaline medium, on the LP (**1**) and LO photodegradation will be presented. Using dielectric spectroscopy, some information about mechanisms of dipole relaxation in the case of the solutions of LP (**1**) in H_2_O and NaOH—reacted LP (**1**) will also be shown. Thus, the dielectric results will provide information regarding the interaction between the water and LP (**1**) (stability of the drug in an aqueous solution), in the absence or presence of NaOH:, for LP:H_2_O samples. The LP (**1**) concentration reduces the interaction energy of water dipoles, while for LP:NaOH samples, the interaction between them leads to a decrease in the characteristic frequency of the dielectric relaxation. 

## 2. Results and Discussion

### 2.1. Photodegradation of LP and the LO drug in Solid State

**Figure 1:**
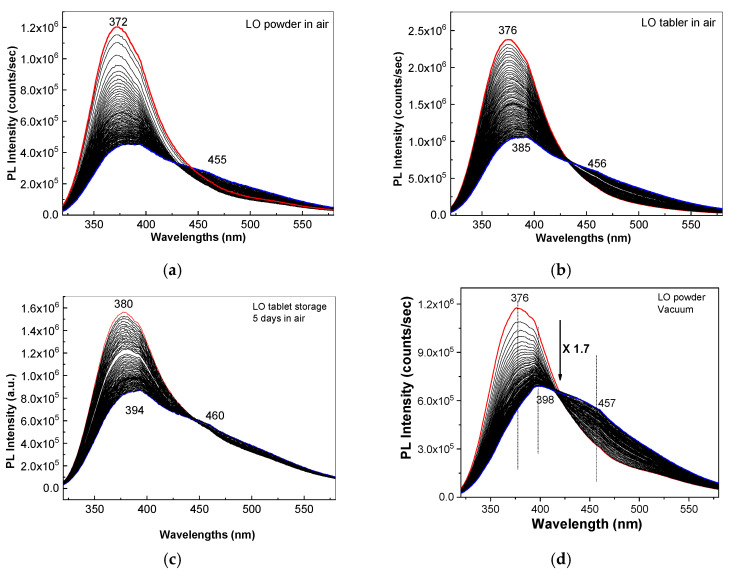
PL spectra of the LO drug in powder state (**a**) and tablet (**b**) and their evolution when the samples are exposed time of 187 min to UV light. The behavior of PL spectra of the LO tablet storage in air, time of 5 days, and their evolution under the UV light exposure (**c**). The influence of the vacuum conditions on the photodegradation of LO in powder state (**d**). In all figures, the red and blue curves correspond to PL spectra in the initial state and after 187 min. of exposure to UV light when the excitation wavelength was of 300 nm. The black curves correspond to the intermediate PL spectra.

**Figure 2:**
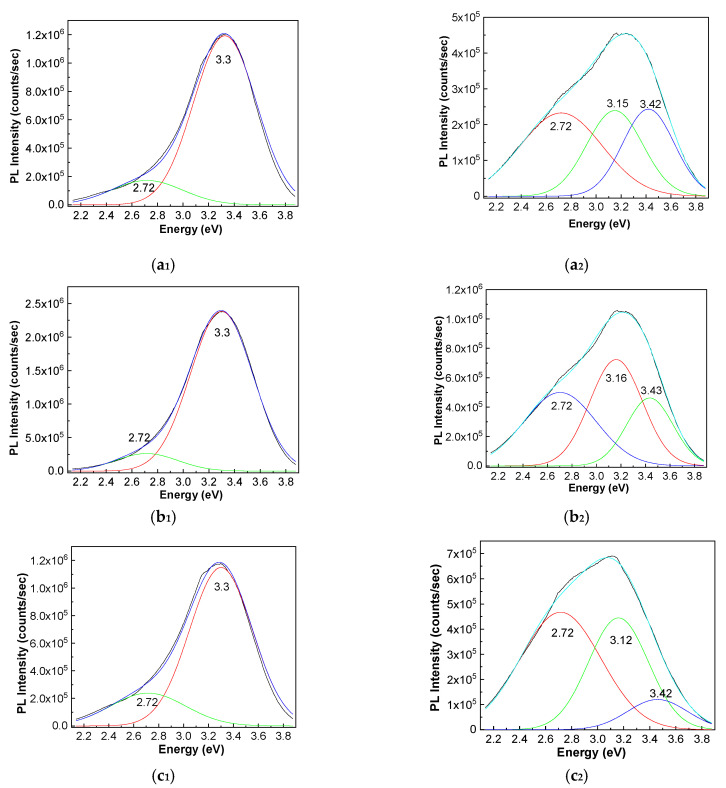
Deconvolution of the PL spectra of the LO drug, in powder state (**a**) and tablet (**b**), before (1) and after the exposure for 187 min. to UV light (2). Figure (**c_1_**_,_**c_2_**) show deconvolution of the PL spectra of LO powder storage 5 days in air, before and after 187 min of UV light exposure.

Figure 1a,b shows the PL spectra of the LO drug, in both powder and tablet form, and its behavior when exposed to UV light. In the initial state, the PL spectra of the LO drug, in powder and tablet form, show an emission band with a maximum of approximately 372–376 nm. The exposure of the LO drug, in both powder and tablet form, to UV light induces: (i) a decrease in the intensity of the PL band, with a maximum of 372–376 nm from 1.2 × 10^6^ counts/sec (red curve in Figure 1a); 2.37 × 10^6^ counts/sec (red curve in Figure 1b) to 4.55 × 10^5^ counts/sec (blue curve in Figure 1a); and 1.05 × 10^6^ counts/sec (blue curve in Figure 1b), respectively; (ii) a shift of the PL band from 372 nm (red curve in Figure 1a) and 376 nm (red curve in Figure 1b) to 383 nm (blue curve in Figure 1a) and 385 nm (blue curve in Figure 1b), respectively; and (iii) the increase in the intensity of the PL band of the LO drug, in powder and tablet form, with the maximum at 455 nm from 2.4 × 10^5^ counts/sec (red curve in Figure 1a) and 4.15 × 10^5^ counts/sec (red curve in Figure 1b) to 2.82 × 10^5^ counts/sec (black curve in Figure 1a) and 6.13 × 10^5^ counts/sec (black curve in Figure 1b), respectively. Figure 2a_1_–b_2_ highlights that, regardless of the form of the LO, i.e., powder or tablet, the following variations are induced by UV light: (i) a shift of the emission band from 3.3 eV to 3.16 eV, which takes place simultaneously with the increase in intensity of the PL band at 2.72 eV; and (ii) the appearance of a new emission band, with the maximum at 3.42 eV. These results clearly indicate that photodegradation of LO occurs under exposure to UV light. 

The storage of the LO tablet in air, five times a day, leads to the following changes in the PL spectra: (a) before UV light exposure: (a_1_) a decrease in the intensity of the emission band, peaking at 376–378 nm from 2.37 × 10^6^ counts/sec (red curve in Figure 1b) to 1.56 × 10^6^ counts/sec (red curve in Figure 1c); (a_2_) a shift of the emission band, from 455 nm (red curve in Figure 1b) to 460 nm (red curve in Figure 1c), which is accompanied by its increase in intensity, from 4.15 × 10^5^ counts/sec (red curve in Figure 1b) to 5.25 × 10^5^ counts/sec (red curve in Figure 1c); and (b) after exposure to UV light, for 187 min, of LO tablet stored for five days in air: (b_1_) a shift of the PL band from 378 nm (red curve in Figure 1c) to 394 nm (blue curve in Figure 1c), variation accompanied by a decrease in this band intensity of approximately 1.8 times, i.e., from 1.56 × 10^6^ counts/sec (red curve in Figure 1c) to 8.68 × 10^5^ counts/sec (blue curve in Figure 1c); and (b_2_) an increase in the intensity of the PL band at 460 nm of 1.08 times is reported. 

Figure 2c_1_–c_2_ highlights the shift of the emission band from 3.3 eV to 3.12 eV, the increase in the intensity of PL band at 2.72 eV and the appearance of the new emission band at 3.42 eV. These variations indicate an amplified degradation of the LO powder whilst being stored in air, evidenced by the higher intensity of the PL band at 2.72 eV. 

In order to highlight the effects of the UV light, in the absence of the water vapors from air, Figure 1d shows the PL spectra of the LO powder, in the vacuum condition, i.e., at a pressure of 7.4 × 10^−6^ mbar. In the latter case, one observes: (i) a decrease in the intensity of the PL spectrum of only 1.71 times, the value of which is lower than the intensity decrease in the case of the PL spectra of the LO drug in powder state (~2.63 times), LO in tablet form (~2.26 times) or the LO sample tablet stored in air for five days (~1.8 times); a shift of the PL band from 376 nm to 398 nm is also reported; and (ii) an increase in the intensity of the emission band at 457 nm of ~1.7 times, a value that is higher than that reported in the case of the LO drug in powder state (~1.4 times), the LO tablet (~1.48 times) or the LO sample stored in air for five days (~1.08 times). These preliminary results clearly indicate the need to store the LO drug and LP under vacuum conditions, in order to reduce the photodegradation processes.

To understand the changes shown in Figure 1 and Figure 2, the potential interactions between the active compounds in the LO drug, i.e., LP (**1**), and the water vapors in the air and the photodegradation processes of excipients used in the pharmaceutical formulation of LO should be taken into consideration. In order to illustrate these aspects, Figure S1, in the Supplementary section, shows the PL spectra of LP (**1**), the excipients of the type of magnesium stearate (MS), SiO_2_, talc, corn starch and cellulose, as well as their blends with LP. According to Figure S1a, the PL spectrum of LP (**1**) shows a band with the maximum at 371 nm, which is asymmetrical in the lower energies range. The exposure of LP (**1**) to UV light leads to the following changes, shown in Figure S1a: (i) a shift of the emission band from 371 nm to 383 nm, with a small variation in intensity from 1.15 × 10^5^ counts/sec to 1.07 × 10^5^ counts/sec; and (ii) an increase in the intensity of the emission band at 457 nm (~2.71 eV) from 3.36 × 10^4^ counts/sec to 7 × 10^4^ counts/sec. Considering these changes, we can conclude that the emission band at ~2.71 eV, reported in the PL spectra of the LO drug exposed to UV light, comes from LP (**1**). 

The influence of various excipients on the LP (**1**) photodegradation is highlighted in Figure S1b_1_–f_2_. Table 1 shows the synthetic variation in terms of the shift of the maxima of PL spectra and the changes in their intensity. 

In a similar manner to the Si quantum dots, the origin of the PL band of SiO_2_ can be explained by the adsorption of water clusters onto SiO_2_ particles [24]. In the case of corn starch (CS), the PL band at 420 nm was also reported by T. Katsumata et al. [25]. The PL spectrum of cellulose (CL) is in good agreement with the study reported by J. Jiang et al., which highlights the excitation wavelengths of 312 nm and 332 nm, an emission band with the maximum at ~400 nm and 440 nm, respectively [26]. 

**Figure 3:**
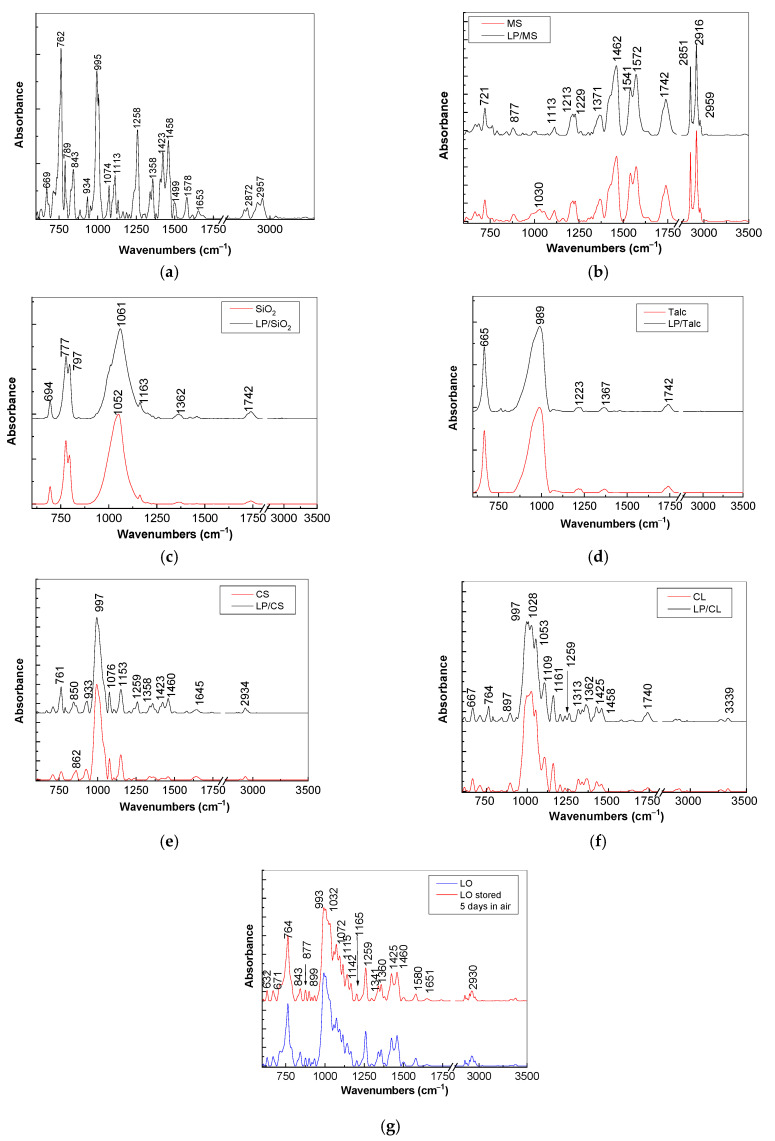
FTIR spectra of the LP (**1**) (**a**) and the blends LP (**1**)/MS (**b**), LP (**1**)/SiO_2_ (**c**), LP (**1**)/talc (**d**), LP(**1**)/C (**e**) and LP(**1**)/CL (**f**). The red curves in FTIR spectra of Figures (**b**–**f**), correspond to the following excipients: MS, SiO_2_, talc, CS and CL. Figure (**g**) shows the FTIR spectra of the LO drug, before and after to storage in air, time of 5 day, under UV light.

Analyzing the above results, we can conclude that, under UV light, an inhibition of the increase of the emission band at ~2.71 eV takes place only in the case of the LP (**1**)/CS and LP (**1**)/CL blends. In order to confirm whether some physical or chemical interactions occur between LP (**1**) and the excipients, the FTIR spectra of these compounds are shown in Figure 3. According to Figure 3a, the IR spectrum of LP (**1**) shows the bands at 762, 789, 843, 934, 995, 1074, 1113, 1258, 1358, 1423, 1458, 1578, 1653, 2872 and 2957 cm^−1^. Table 2 shows the vibrational modes of the IR bands shown in Figure 3a. 

Figure 3b–f highlight: (i) that, in the case of the LP (**1**)/MS and LP (**1**)/talc blends, there are no major changes compared to the excipients alone (Figure 3b,d); (ii) in the case of the LP (**1**)/SiO_2_ blend, there was a down-shift of the IR band from 1061 cm^−1^ to 1052 cm^−1^ and an increase in the absorbance of the IR bands, which peaked at 1163, 1362 and 1742 cm^−1^ (Figure 3c); the IR bands at 1061, 1163, 1362 and 1742 cm^−1^ of SiO_2_ are assigned to vibrational modes, shown in Table 3. (iii) in the case of the LP (**1**)/CS blend, an increase in the absorbance of the IR bands peaked at 761, 1259, 1358, 1423 and 1460 cm^−1^ as well as a down-shift of the IR band from 862 to 850 cm^−1^, without other changes in the position or profile of the IR band at 997 cm^−1^ (Figure 3e). The IR bands of CS at 761, 862, 1259, 1358, 1423 and 1460 cm^−1^ are assigned to vibrational modes, shown in Table 3; (iv) in the case of the LP (**1**)/CL blend, a change in the ratio between the absorbance of the IR bands peaked at 997 and 1028 cm^−1^, as well as the absorbance growth of the IR bands, which peaked at 764, 1259, 1425, 1458 and 1740 cm^−1^. The IR bands of CS at 764, 997–1028, 1259, 1425–1458 and 1740 cm^−1^ are assigned to vibrational modes, shown in Table 3. The variations reported in the case of the LP (**1**)/SiO_2_, LP (**1**)/CS and LP (**1**)/CL blends indicate that excipients SiO_2_, CS and CL facilitate the faster adsorption of water from the air, thus accelerating the photodegradation of the drug. 

**Scheme 1:**
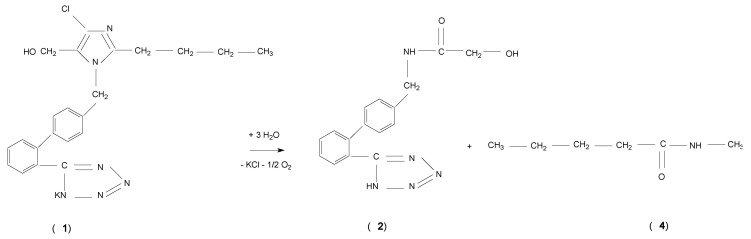
The interaction of LP with water vapors from air.

The main vibrational changes, induced by the storage of the LO drug in air for five days, are shown in Figure 3g through FTIR spectroscopy. The storage of the LO drug in air induces in the FTIR spectra an increase in the absorbance of the IR bands, which peaked at 1032, 1072, 1115 and 1142 cm^−1^; these are positioned close to those previously reported at 1032 cm^−1^ in CL or 1030 cm^−1^ in LP (**1**) [13]; 1072 cm^−1^ and 1113 cm^−1^ in LP; and 1153 cm^−1^ in CS. The IR band at 1030 cm^−1^ in LP (**1**) was assigned to the vibrational modes of C-O stretching, C-Cl stretching and C-C-C bending in the aromatic rings and N-N-N bending in the tetrazole ring [13]. The variations reported in Figure 3g indicate that the interaction of LP (**1**) with water vapors from air induces changes in the vibrational modes of the CH_2_ bond [31,32] or C-O stretching, C-C stretching, C-C bending and N-N-N bending in the tetrazole ring of LP(**1**) [13], C-N-C bending in the imidazole ring and C-O stretching, C-Cl stretching [12], and C-H wagging in the benzoic ring [13] and C-O stretching [30]. These changes can be explained by taking into account Scheme 1, which shows the interaction of LP (**1**) with water vapors from the air. Scheme 1 shows that the reaction products correspond to the following organic compounds: N-methanolamide-{[2′-(1H-tetrazol-5-yl)(1,1′-biphenyl)-4-yl]methyl} (**2**), N-methyl pentanamide (**4**), potassium chloride (KCl) and oxygen (O_2_). 

According to Scheme 1, the increase in the absorbance of the IR band at 1032 cm^−1^ and 1072 cm^−1^ is a result of the appearance of new C-O bonds in the photodegradation products of LP(**1**). To summarize the above results, we can conclude that: (i) the photodegradation process of the LO drug is caused by the interaction of LP (**1**) with water vapors in the air; and (ii) the excipients CS, CL and SiO_2_ contribute to the photodegradation of the LO drug as a result of the appearance of new hydrogen bonds. In order to avoid these inconveniences, the LO drug should be packed into aluminum blisters under vacuum; this measure could prevent the photodegradation of this drug. 

### 2.2. The Photodegradation of the LO Drug in the Presence of PBS 

**Figure 4:**
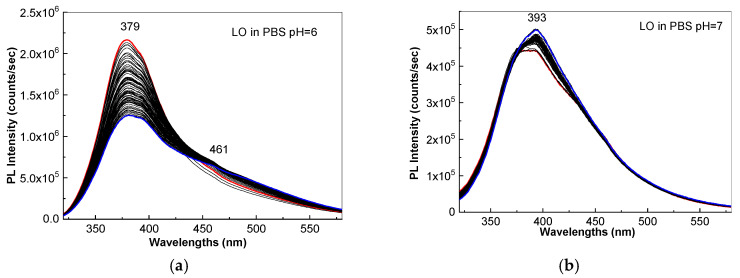
PL spectra of the LO drug in PBS having pH equal to 6 (**a**) and 7 (**b**), when the excitation wavelength is 300 nm. The red, black and blue curves correspond to the PL spectra of samples before, in the intermediate state and after 187 min. of exposure to UV light.

Figure 4 shows the PL spectra of LO in PBS, with pH equal to six and seven.

According to Figure 4, depending on the PBS pH value, i.e., six or seven, the intensity of the PL spectra of the LO drug, before to exposure to UV light, varies from 2.16 × 10^6^ counts/sec to 4.48 × 10^5^ counts/sec, the peak of the emission bands being localized at 379 and 388 nm, respectively. The exposure to UV light for 187 min leads to: (i) a decrease in the intensity of the PL band of the LO drug in PBS, with pH=6 to 1.25 × 10^5^ counts/sec, the emission peak being localized at 382 nm; and (ii) an increase in the intensity of the PL band of the LO drug in PBS, with pH = 7 to 4.99 × 10^5^ counts/sec, the emission band maximum being shifted to 393 nm. Regardless of the PBS pH value, the profile of the PL spectra of the LO drug are asymmetric in the low energies range as a result of the emission band at 460 nm (Figure 4). As we will demonstrate in the following section, the PL band maximum, at 393 nm, is characteristic for the LO solution in the presence of the alkaline media.

### 2.3. Photodegradation of LO and LP in the Presence of Alkaline Media

**Figure 5:**
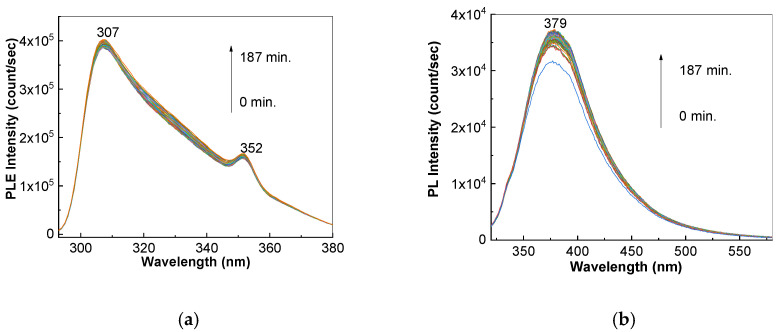
PLE (**a**) and PL (**b**) spectra of the LP aqueous solution, under UV light exposure for 187 min.

Before showing the PL spectra of LO and LP (**1**) in the presence of alkaline media, the photoluminescence excitation (PLE) and PL spectra of the LP aqueous solution will be shown. Figure 5 demonstrates that: 

(i) the PLE spectrum of LP (**1**) shows a band at 307 nm with an intensity of 4 × 10^5^ counts/sec, and this band is accompanied by another band of low intensity, at 352 nm; and

(ii) the PL band of LP (**1**) shows a maximum at 379 nm, with an intensity equal to 3.2 × 10^4^ counts/sec. 

The exposure of LP to UV light does not induce significant changes to the PLE spectrum, while in the case of the PL spectra, an intensity increase of up to 3.6 × 10^4^ counts/sec is observed.

**Figure 6:**
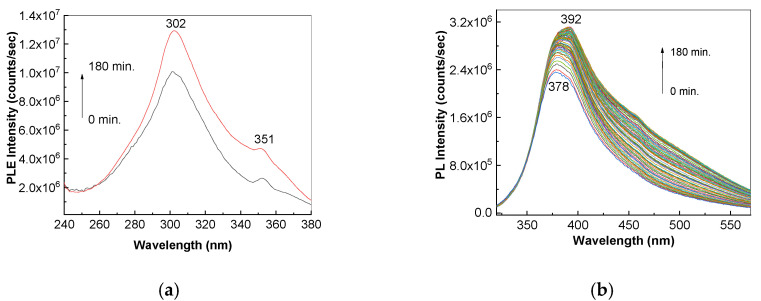
PLE (**a**) and PL (**b**) spectra of the LO aqueous solution and their evolution under UV light.

Figure 6a,b highlights the PLE and PL spectra of LO when in dark conditions. From this figure, one can observe: (i) a band at 302 nm, with the intensity of 1 × 10^7^ counts/sec, in the PLE spectrum; and (ii) an emission band, at 378 nm, with the intensity of 2.35 × 10^6^ counts/sec, in the PL spectrum. Following the exposure of LO to UV light, the intensity of the PLE and PL spectra increase up to 1.29 × 10^7^ counts/sec and 3.1 × 10^6^ counts/sec, respectively; this modification is accompanied by a shift in the PL band, from 378 nm to 392 nm, simultaneously, with a variation of the bandwidth resulting from an increase in the intensity of the luminescent center at 456 nm.

**Figure 7:**
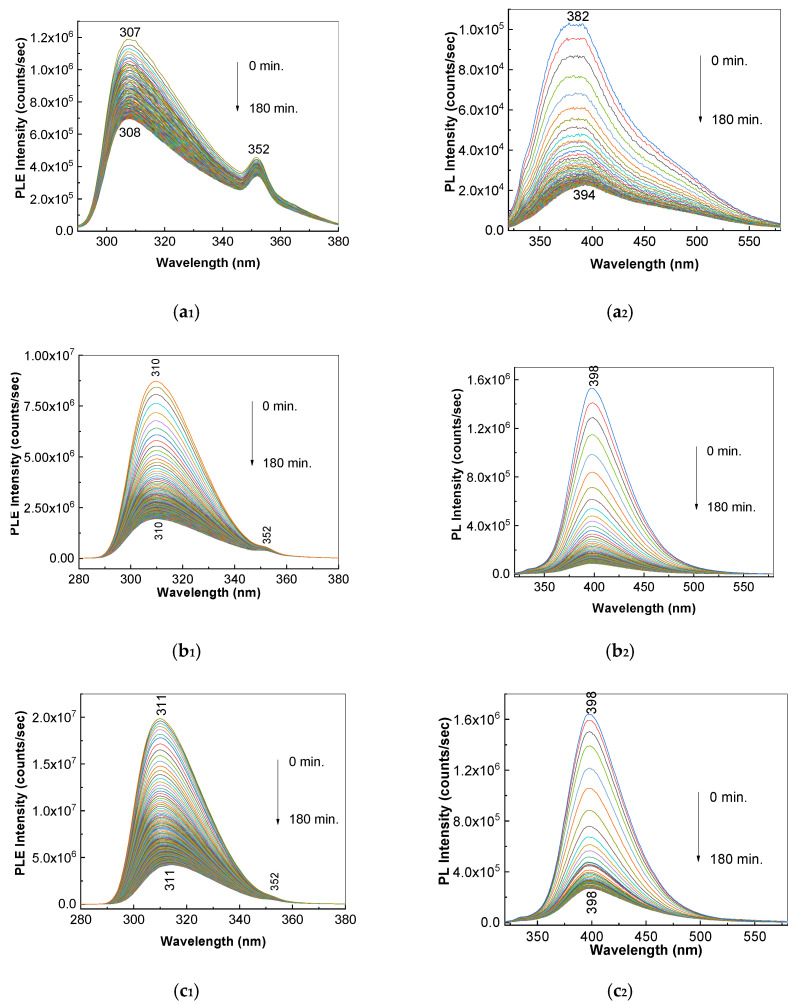
PLE and PL spectra of NaOH-reacted LP (**1**), the volumetric ratio of the LP (**1**) and NaOH is equal to: 2:1 (**a_1_**,**a_2_**), 1.5:1.5 (**b_1_**,**b_2_**) and 1:2 (**c_1_**,**c_2_**), and their evolution under UV light.

**Figure 8:**
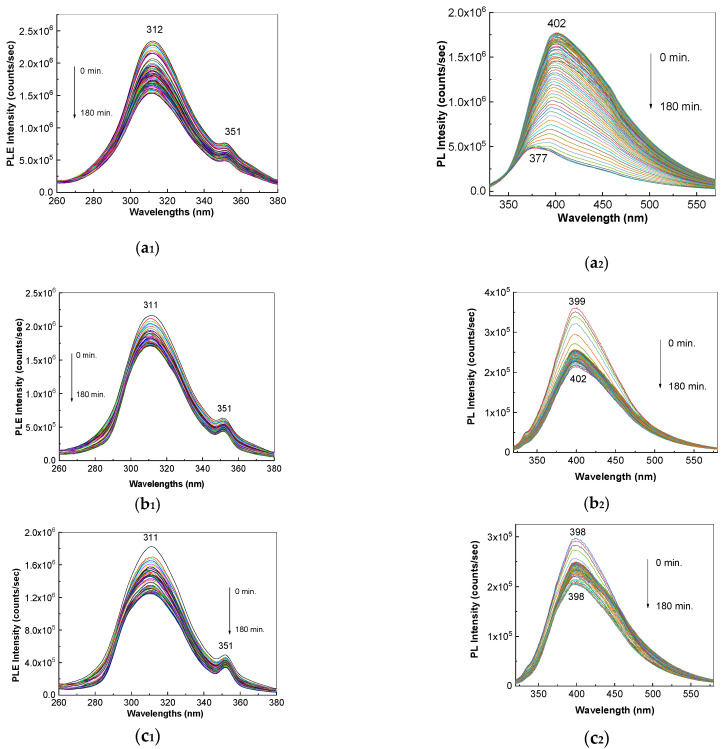
PLE and PL spectra of NaOH-reacted LO, the volumetric ratio of the LO and NaOH is equal to 2:1 (**a_1_**,**a_2_**), 1.5:1.5 (**b_1_**,**b_2_**) and 1:2 (**c_1_**,**c_2_**) and their evolution when the samples are exposed to UV light.

With this in the mind, Figure 7 and Figure 8 show the PLE and PL spectra of NaOH-reacted LP (**1**) and LO, respectively. Thus, the NaOH-reacted LP (**1**) highlights: (i) in dark conditions, an intensity growth of PLE band from 1.19 × 10^6^ (Figure 7a_1_) to 8.71 × 10^6^ counts/sec (Figure 7b_1_) and 1.98 × 10^7^ counts/sec (Figure 7c_1_), simultaneous with the intensity increase of the PL spectrum from 1.02 × 10^5^ counts/sec (Figure 7a_2_) to 1.53 × 10^6^ counts/sec (Figure 7b_2_) and 1.64 × 10^6^ counts/sec (Figure 7c_2_). These changes are accompanied by a shift of the PLE and PL bands from, 307 nm and 379 nm (Figure 5) up to 310 nm and 398 nm, respectively (Figure 7). These variations indicate the emergence of a new luminescent center in the case of NaOH-reacted LP(**1**). The effect of UV light on the three samples of NaOH-reacted LP (**1**), time of 3 h, consists of an intensity decrease in both (i) the PLE spectrum, i.e., up to 6.98 × 10^5^ counts/sec (Figure 7a_1_) to 1.95 × 10^6^ counts/sec (Figure 7b_1_) and 4.22 × 10^6^ counts/sec (Figure 7c_1_), and; (ii) the PL spectrum, namely up to 2.3 × 10^4^ counts/sec (Figure 7a_2_), 8.84 × 10^4^ counts/sec (Figure 7b_2_) and 2.65 × 10^5^ counts/sec (Figure 7c_2_).

The reaction of LO with NaOH leads: 

(i) in the dark conditions, to a progressive diminution of the PLE spectrum intensity up to 2.36 × 10^6^ counts/sec (Figure 8a_1_), 2.17 × 10^6^ counts/sec (Figure 8b_1_) and 1.84 × 10^6^ counts/sec (Figure 8c_1_), simultaneous with an intensity decrease of the PL spectra, up to 1.76 × 10^6^ counts/sec (Figure 8a_2_), 3.6 × 10^5^ counts/sec (Figure 8b_2_) and 2.96 × 10^5^ counts/sec (Figure 8c_2_), when the volumetric ratio of LO:NaOH is 2:1, 1.5:1.5 and 1:2, respectively; these changes are accompanied by a variation in the PL spectrum maximum at 402 nm (Figure 8a_2_), 399 nm (Figure 8b_2_) and 398 nm (Figure 8c_2_) and (ii) after UV irradiation, to a variation in the PLE and PL spectra intensity, as well as the PLE spectra profile. Thus, when the volumetric ratio of LO:NaOH is equal to: (a) 1:2, a decrease of the PLE spectrum intensity from 1.84 × 10^6^ counts/sec to 1.27 × 10^6^ counts/sec occurs (Figure 8c_1_); (b) 2:1 and 1.5:1.5, a variation in the PLE spectra intensity takes place from 2.36 × 10^6^ counts/sec (Figure 8a_1_) and 2.17 × 10^6^ counts/sec (Figure 8b_1_) to 1.53 × 10^6^ counts/sec (Figure 8a_1_) and 1.71 × 10^6^ counts/sec, respectively (Figure 8b_1_); and (c) 2:1, 1.5:1.5 and 1:2, the PL spectrum intensity varies from 1.76 × 10^6^ counts/sec (Figure 8a_2_), 3.6 × 10^5^ counts/sec (Figure 8b_2_) and 2.96 × 10^5^ counts/sec (Figure 8c_2_) to 4.81 × 10^5^ counts/sec (Figure 8a_2_), 2.14 × 10^5^ counts/sec (Figure 8b_2_) and 2.06 × 10^5^ counts/sec (Figure 8c_2_). 

Summarizing these results, they indicate that: (i) the NaOH has the role of the LP (**1**) PL quenching agent; and (ii) the UV irradiation of the NaOH-reacted LP (**1**) induces a photodegradation process, remarked by an additional intensity decrease of the PLE and PL spectra.

**Figure 9:**
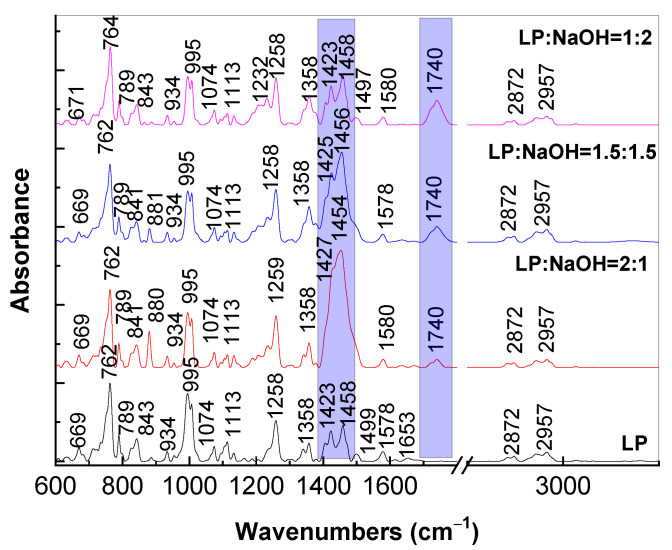
IR spectra of LP (**1**) (**black curve**) and NaOH-reacted LP (**1**) when the volumetric ratio LP:NaOH is 2:1 (**red curve**), 1.5:1.5 (**blue curve**) and 1:2 (**magenta curve**).

To explain these variations, Figure 9 shows the IR spectra of the LP (**1**) and the NaOH-reacted LP (**1**). The reaction of LP (**1**) with NaOH is proven by the following changes in Figure 9: (i) the appearance of the IR band at 1740 cm^−1^, which indicates the generation of a degradation product, containing the functional groups with the C=O bonds [33,34], whose intensity increases with the NaOH volume in the LP:NaOH mixture; (ii) a variation of the ratio between the IR bands absorbance peaked at (a) 995 cm^−1^ and 1454–1458 cm^−1^; and (b) 1258–1259 cm^−1^ and 1454–1458 cm^−1^, from 1.9 and 1.14 (Figure 9, black curve) to 0.47 and 0.44 (Figure 9, red curve), 0.57 and 0.58 (Figure 9, blue curve), as well as 0.95 and 0.92 (Figure 9, magenta curve); and (iii) a decrease of the ratio between the IR bands absorbance, localized at 1423–1427 cm^−1^ and 1454–1458 cm^−1^, associated to the vibrational modes: (i) C-N stretching in the tetrazole ring and C-H wagging at the imidazole ring and C-H scissor between the benzoic and the imidazole rings and (ii) C-O-H bending and C-H scissor in the alkyl chain, respectively, from 0.83 (Figure 9, red curve) to 0.77 (Figure 9, blue curve) and 0.76 (Figure 9, magenta curve), values which are smaller than that reported for LP (**1**) (0.86 in Figure 9, black curve).

**Scheme 2:**
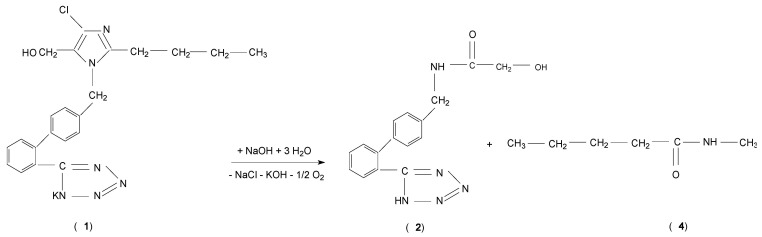
The photodegradation reaction of LP (**1**) in the presence of the NaOH aqueous solution.

Considering the changes discussed above, the photodegradation reaction of the NaOH-reacted LP (**1**) can be described according to Scheme 2: the reaction products correspond to N-methanolamide-{[2′-(1H-tetrazol-5-yl)(1,1′-biphenyl)-4-yl]methyl} (**2**), N-methylpentanamide (**4**), sodium chloride (NaCl), potassium hydroxide (KOH) and oxygen (O_2_). At this stage of our study, according to the 10th edition of the Ph. Eur. and References [19,20], both LP and the products of the photodegradation reactions highlighted in Scheme 1 and Scheme 2, could also lead to nitrosamine impurities. However, no nitrosamine impurities were detected in the present study.

In our opinion, the first photodegradation product invoked in Scheme 2, i.e., (2), is sustained by the IR bands at 1423–1427 and 1740 cm^−1^, which were attributed to the functional groups C=O and −OH, respectively. 

To emphasize the importance of the studies presented in Section 2.2 and Section 2.3, we highlight that, at present, there are more sensors for the electrochemical detection of LO or LP (**1**) in synthetic human body fluids, which involve the use of PBS with pH ranging between 2.0 and 7.0 [35] or the Britton-Robinson buffer, with a pH equal to 9.5 [10]. So far, to the best of the authors’ knowledge, a study on LO and LP (**1**) detection that also takes into account the effect of UV light, from the stage of sample preparation or collection of biological fluids to the actual measurements of the determination of the concentration of active compounds in pharmaceutical formulations, has not been reported. Considering the results shown in this work, it is necessary to study the UV light effect when the pharmaceutical compound detection is carried out by the electrochemical or optical analytical methods.

### 2.4. Mechanisms of Dipole Relaxation of LP (1) in H_2_O and NaOH, Respectively

**Figure 10:**
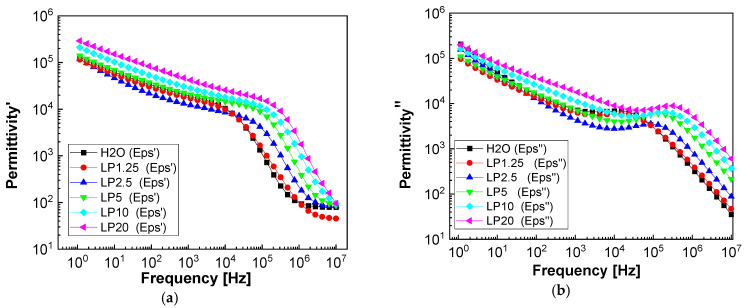
The dependence of the dielectric constant (**a**) and dielectric loss (**b**) with the frequency, in the range of the LF, when the concentration of the LP (**1**) solution varies from 0 (black curve) to 0.125 mg/mL (red curve), 0.25 mg/mL (blue curve), 0.5 mg/mL (green curve), 1 mg/mL (cyan curve) and 2 mg/mL (magenta curve).

**Figure 11:**
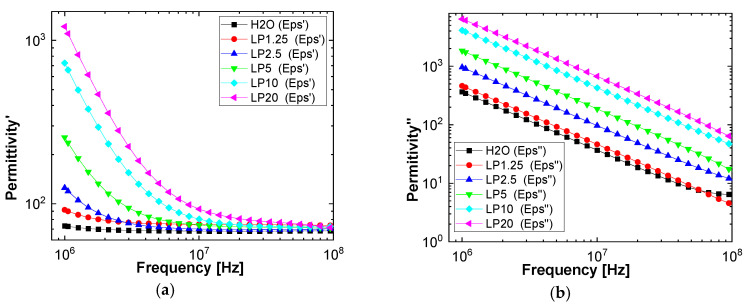
The dependence of the dielectric constant (**a**) and dielectric loss (**b**) with the frequency, in the range of the HF, when the concentration of the LP (**1**) solution varies from 0 (black curve) to 0.125 mg/mL (red curve), 0.25 mg/mL (blue curve), 0.5 mg/mL (green curve), 1 mg/mL (cyan curve) and 2 mg/mL (magenta curve).

**Figure 12:**
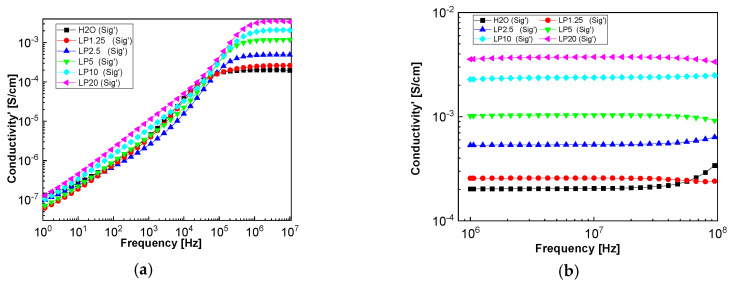
The dependence of real part of conductivity with frequency, in the range of low (**a**) and high (**b**) frequencies, when the concentration of the LP(**1**) solution varies from 0 (black curve) to 0.125 mg/mL (red curve), 0.25 mg/mL (blue curve), 0.5 mg/mL (green curve), 1 mg/mL (cyan curve) and 2 mg/mL (magenta curve).

**Figure 13:**
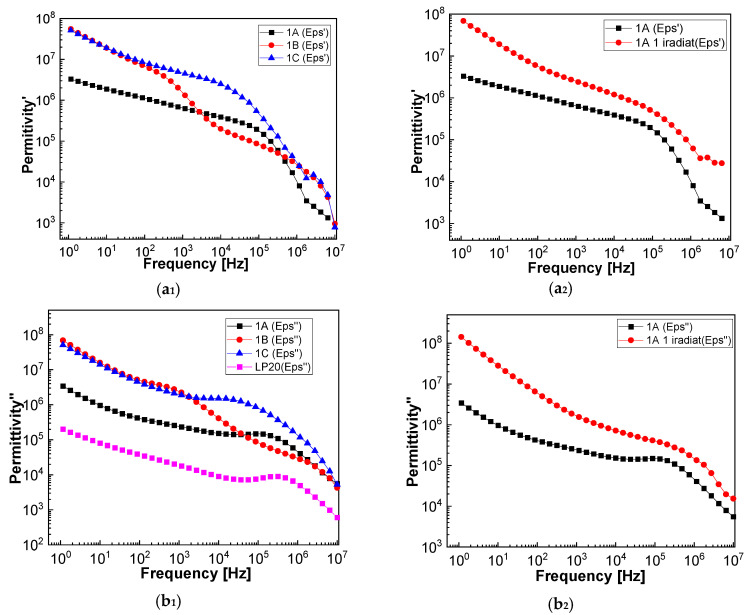
The dependence of the dielectric constant and dielectric loss with the frequency, in the LF range, in the case of the 1A, 1B and 1C samples as well as LP (**1**) before to exposure to UV light (**a_1_**,**b_1_**). (**a_2_**,**b_2_**) show the dependence of the dielectric constant and dielectric loss with the frequency in the case of the 1A sample before and after exposure to UV light (labelled as 1A1).

**Figure 14:**
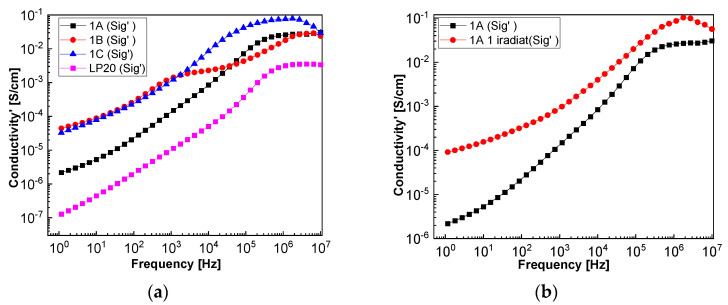
The dependence of real part of conductivity with frequency, in the range of low frequencies, in the case of the 1A, 1B and 1C samples as well as LP (**1**) before to exposure to UV light (**a**). (**b**) shows the dependence of the real part of conductivity with frequency in the case of the 1A sample before and after exposure to UV light, labelled as 1A1.

In order to describe the nature of the interaction between LP and water, in the absence and in the presence of NaOH, Figure 10, Figure 11, Figure 12, Figure 13 and Figure 14 show the dependence of the real (dielectric constant, ε’) and imaginary part (dielectric loss, ε”) of the permittivity and the real part of conductivity with frequencies, in the low (LF) and high (HF) frequencies range.

Figure 10 and Figure 11 present the frequency-dependent spectra of the real and imaginary part of complex permittivity for all of the investigated samples, at room temperature, in the 0.1–100 MHz range. From these figures, it can be seen that the values of the real and imaginary parts of the dielectric constant decrease with the increase in frequency. At low frequency, between 10 kHz–1 MHz, the dielectric constant has a step variation (Figure 10a), while the dielectric loss has a maximum point (Figure 10b). These aspects show a H_2_O molecular electric dipolar relaxation. The frequency of the maximum point increases with the increase in the concentration of the LP (**1**). This could result from the decrease of the dipolar interaction energy of H_2_O molecules. The maximum dielectric loss point represents the characteristic frequency. In addition, at high frequency, over 10 MHz, ε’ tends to be an independent value of frequency, on the graph being a plateau (in Figure 11a). Instead, the dielectric loss varies continuously with frequency (Figure 11b). Very high ε’ and ε” values at low frequencies indicate the presence of the charge carriers, which induce an electrode polarization effect. The occurrence of the plateau, i.e., almost constant values of ε’ at frequencies higher than 10 MHz, indicates that the polarization effects due to electrical conduction charges are suppressed by the dielectric polarization effects [36]. An important remark is that the values of dielectric constant and dielectric loss increase with increasing the LP (**1**) concentration (Figure 10 and Figure 11). The conductivity spectra (Figure 12) show the same dependence of the values with the concentration of LP (**1**), a result confirmed by the variation of the permittivity components. 

In Figure 13 and Figure 14, the dependence of the dielectric constant and dielectric loss, as well as the real part of conductivity with frequencies, are shown in the case of: (a) the 1A, 1B and 1C samples, which correspond to the LP:NaOH volumetric ratio equal to 2:1, and 1:2, respectively, and the aqueous solution of NaOH 1.5 M; and (b) the 1A1 sample, that resulted from the 1A sample being exposed to UV light for 187 min.

Through the volumetric ratio modification of the sample (1A and 1B), the values and aspect of the permittivity and dielectric loss spectra are changed (Figure 13a_1_,b_1_). From Figure 13b_1_, it can be observed that the characteristic frequency decreases with the increasing NaOH concentration. This can be explained by the interaction between the two components of the samples. The spectra of LP (**1**) were introduced in the graph (Figure 13b_1_) as a reference for pure material (this sample has the largest amount of LP (**1**)).

UV irradiation significantly modifies the electrical properties of the samples (Figure 13a_2_,b_2_), leading to values that are almost two orders of magnitude larger than the permittivity components. The same effect is also observed of the conductivity behavior (Figure 14b).

Conductivity increases with the increasing NaOH concentration (Figure 14a). The spectra of LP were introduced in the graph (Figure 14a) as a reference for pure material. The significant decrease in conductivity at low frequencies is due to the polarization effects at the electrode (Figure 12a and Figure 14a,b). 

## 3. Materials and Methods

LP (**1**), MS, CS, CL, SiO_2_, talc and NaOH were from the Aldrich-Sigma company. The drug marked under the name LO was from a local pharmacy. The composition of the LO drug consists of 50 mg LP, a core containing pregelatinized starch, anhydrous colloidal SiO_2_, corn-starch, celactose (cellulose and lactose monohydrate), magnesium stearate and a film made from Hypromellose, propylene glycol, TiO_2_ and talc.

The LP (**1**) aqueous solutions with concentration of 5 mg/mL and NaOH 1.5 M were prepared. The reaction of these chemical compounds was achieved using an equal volumetric ratio of the LP (**1**) and NaOH, with 2:1, 1.5:1.5 and 1:2. The pH value of the 5mg/mL LP solution is equal to seven, while the pH of the LP samples interacted with NaOH; when the volumetric ratio of the two constituents is 2:1, 1.5:1.5 and 1:2, this varies from 9 to 9.4 and 9.8, respectively. To prepare a LO aqueous solution, the grounding of a tablet was achieved and, then, the powder was dispersed in 10 mL H_2_O and ultrasonicated for 20 min. Subsequently, the suspension was filtered to obtain a clear solution.

The blends involving LP (**1**) and excipients of the type MS, CS, CL, SiO_2_ and talc were prepared by grinding, the mass ratio being 1:3 (w/w).

The photoluminescence (PL) and photoluminescence excitation (PLE) spectra of the LP (**1**) and LO aqueous solutions were registered with a Fluorolog spectrometer, FL3-22 model, from Horiba Jobin Yvon, in a right-angle (RA) geometry, using a Xe lamp with a power of 450 W as the excitation source. The excitation and emission wavelengths for the recording of the PL and PLE spectra were equal to 300 and 400 nm, respectively. At the end of the PL studies, the samples were recovered and analyzed by FTIR spectroscopy and dielectric spectroscopy.

The IR spectra of LP, before it reacted with NaOH, was registered in the attenuated total reflection (ATR) geometry, with a Vertex 70 FTIR spectrophotometer, from Bruker, endowed with a MKII Golden Gate TM single reflection ATR system. The recording of the IR spectra was carried out with a resolution is of 3 cm^−1^, the scans number being equal to 64. 

The broad band dielectric spectroscopy (DS) method was applied using Alpha-A High Performance Frequency Analyzer from NOVOCONTROL GmbH, for a low frequency range (0.1 Hz–10 MHz), as well as a RF Impedance Analyzer Agilent E4991A at high frequency (1 MHz–100 MHz), in order to investigate the electrical properties of the samples as function of the frequency at room temperature. The method is based on the interaction between the externally applied electric field and the charge carriers in the sample. The sample is placed in a plan parallel capacitance configuration using circular metallic electrodes.

## 4. Conclusions

In this manuscript, we have reported new results regarding the degradation processes of LO and LP (**1**), induced by UV light, the PBS pH and alkaline medium. Our results have highlighted that: (i) the photodegradation of LO, in solid form, is caused by the degradation of LP in the presence of water vapors from air and UV light; (ii) according to the PL and FTIR studies, the excipients CS, CL and SiO_2_ contribute to the photodegradation of the LO drug; (iii) in dark conditions, the reaction of LP (**1**) with NaOH leads to an enhancement of the PL spectra intensity, from the spectral range 320–580 nm, which has indicated the emergence of a new luminescent center; (iv) the photodegradation of NaOH-reacted LP (**1**) or LO induces a decrease in the PLE and PL spectra intensity; this fact was explained as a result of the generation (**2**) and (**4**); (v) the emergence of the new IR band at 1740 cm^−1^ and the higher absorbance of the IR band at 1423–1427 cm^−1^ indicate C_6_H_15_N_5_O_2_ to be the main photodegradation product; and (vi) according to the dielectric spectroscopy, in the case of the samples LP:H_2_O and LP:NaOH, of various concentrations, over a broad range of frequency, 0.1 Hz to 100 MHz, at room temperature, an electrode polarization phenomenon has been observed. The mechanisms of dipole relaxation are influenced by the composition of the samples. Thus, in the sample made up of LP (**1**) and water, the LP (**1**) concentration reduced the interaction energy of the water dipoles. In the case of the LP (**1**) and NaOH samples, the interaction between them led to a decrease in the characteristic frequency of the dielectric relaxation. The conductivity and permittivity components show the same dependence with the LP (**1**) concentration. The electrical properties of the samples are significantly modified by irradiating them with UV light, thus obtaining values that are almost two orders of magnitude higher than the permittivity components of the non-irradiated sample.

## Figures and Tables

**Table 1:** The main variations of PL spectra of excipients and their mixture with LP (**1**) during to exposure to UV light.

Excipient	Blends Based on LP (1) and Excipient	The Range of Changes of PL Bands (nm)	∆I^PL^ = $|I_{t=187}^{PL}$$-I_{t=0}^{PL}|$(Counts/Sec)	Time of Exposure to UV Light (min)
MS	-	435–452	1.78 × 10^4^	187
MS	LP (**1**)/MS	395	4.54 × 10^5^	187
MS	LP (**1**)/MS	449	0.54 × 10^5^	187
SiO_2_	-	374–393	2.14 × 10^4^	187
SiO_2_	LP (**1**)/SiO_2_	375–380	1.24 × 10^4^	187
SiO_2_	LP (**1**)/SiO_2_	460	2.73 × 10^3^	187
Talc	-	440–450	2.45 × 10^4^	187
Talc	LP (**1**)/Talc	381–385	0.33 × 10^4^	187
Talc	LP (**1**)/Talc	446	0.48 × 10^4^	187
CS	-	420–427	0.6 × 10^5^	187
CS	LP (**1**)/CS	460	0.5 × 10^4^	187
CL	-	402–440	0.24 × 10^5^	187
CL	LP (**1**)/CL	378	1.43 × 10^4^	187
CL	LP (**1**)/CL	460	0.48 × 10^4^	187

**Table 2:** The IR bands of LP (**1**) and their vibrational modes.

Wavenumbers (cm^−1^)	Vibrational Modes Assigned [13]
762	C-H wagging at benzoic ring and alkyl chain
789	C-H rocking in alkyl chain
843	C-H wagging at benzoic ring
934	C-N-C bending in imidazole ring and C-H rocking in alkyl chain and C-H torsion in alkyl chain
995	N-C-N bending in first ring and C-C bending in the second ring
1074	C-N-C bending in imidazole ring and C-O stretching and C-Cl stretching
1113	C-H wagging at benzoic ring
1258	C-N stretching in imidazole ring and C-C stretching between benzoic ring and benzoic rings and C-H wagging at benzoic rings and in the alkyl chain
1358	C-H wagging benzoic and imidazole ring
1423	C-N stretching in tetrazole ring and C-H wagging at imidazole ring and C-H scissor between benzoic and imidazole ring and C-O-H bending
1458	C-H scissor in the alkyl chain
1578	C=C stretching in imidazole ring and C-C stretching in benzoic rings
1653	C-C stretching in benzoic ring and C-H wagging
2872	C-H stretching at imidazole ring
2957	C-H stretching in alkyl chain

**Table 3:** The main IR bands of excipients of LO and their assignments.

Excipient	Wavenumbers (cm^−1^)	Vibrational Mode	References
SiO_2_	1061	Si-O symmetrical stretching in O-Si-O,	[27,28]
SiO_2_	1163	Si-O asymmetrical stretching in O-Si-O,	[27,28]
SiO_2_	1362	CH_3_	[27,28]
SiO_2_	1742	C=O	[27,28]
CS	761	bending for the CH_2_ group	[29,30]
CS	862	anhydro-glucose ring	[29,30]
CS	1259	CH_2_,	[29,30]
CS	1358	CCH bending	[29,30]
CS	1423	OH bonds	[29,30]
CS	1460	CH bonds	[29,30]
CL	764	CH_2_,	[31,32]
CL	997–1028	C-O bond stretching	[31,32]
CL	1259	C-CO/O-H in-plan deformation	[31,32]
CL	1425–1458	CH_2_ symmetric bending	[31,32]
CL	1740	C=O stretching	[31,32]

## Data Availability

Data is contained within the article.

## Supplementary Materials

The following supporting information can be downloaded at: https://www.mdpi.com/article/10.3390/pharmaceutics14112419/s1, Figure S1: PL spectra of the LP (a), MS (b_1_), SiO_2_ (c_1_), talc (d_1_), corn starch (e_1_) and cellulose (f_1_) and their blend with LP, i.e., LP/MS (b_2_), LP/SiO_2_ (c_2_), LP/talc (d_2_), LP/corn starch (e_2_) and LP/cellulose (f_2_) as well as their evolution when the samples are exposed to UV light, for 187 min. All PL spectra are recorded at the excitation wavelength of 300 nm. The red, black and blue curves correspond to the PL spectra of samples before, in the intermediate state and after 187 min. of UV light exposure.
