# Supplementary material for: Degradation of Losartan Potassium Highlighted by Correlated Studies of Photoluminescence, Infrared Absorption Spectroscopy and Dielectric Spectroscopy
Source: Pharmaceutics. 2022 Nov 9;14(11):2419. doi: 10.3390/pharmaceutics14112419 (PMC9692865; doi:10.3390/pharmaceutics14112419)
Supplement: Supplementary file 1 [file pharmaceutics-14-02419-s001.zip › pharmaceutics-1833649-supplementary.pdf]

Supplementary part

Degradation of Losartan Potassium Highlighted by Correlated Studies of Photoluminescence, Infrared Absorption Spectroscopy and Dielectric Spectroscopy

Mirela Paraschiv [1,2], Ion Smaranda[1], Irina Zgura[1], Paul Ganea[1], Madalina Chivu[1], Bogdan Chiricuta[3] and Mihaela Baibarac[1,*]

[1] National Institute of Materials Physics, Laboratory of Optical Processes in Nanostructured Materials, Atomistilor street 405A, P.O.Box MG-7, R077125, Bucharest, Romania
[2] Univ. Bucharest, Fac. Phys, POB MG-11, Bucharest 077125, Romania
[3] SC Apel Laser SRL, 25 Vanatorilor St, Mogosoaia 077135, Romania
* Correspondence: barac@infim.ro; Tel.: +40-21-3690170

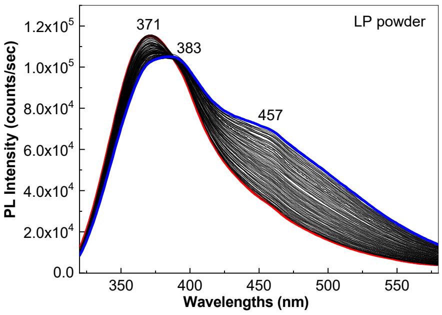

(**a**)

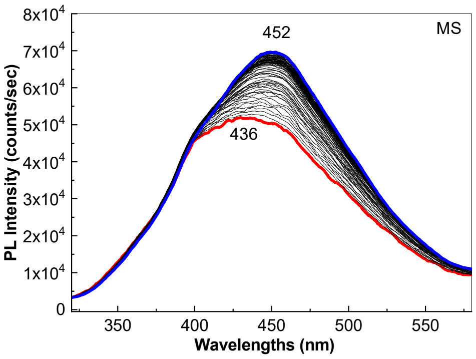

(**b1**)

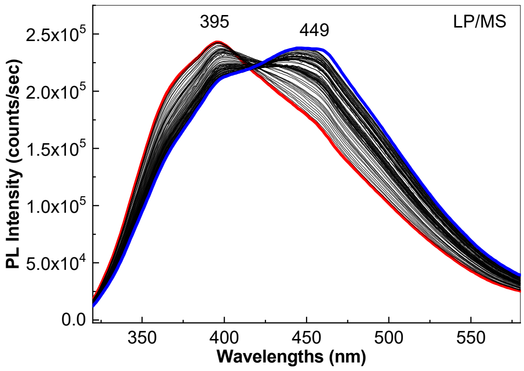

(**b2**)

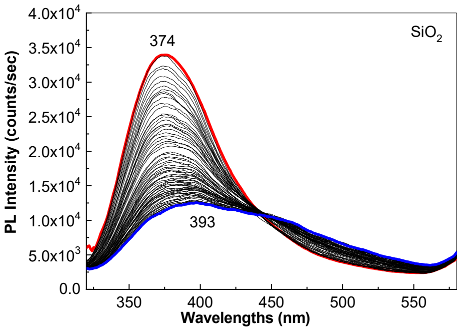

(**c1**)

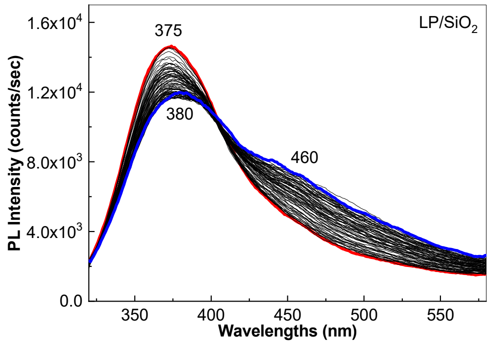

(**c2**)

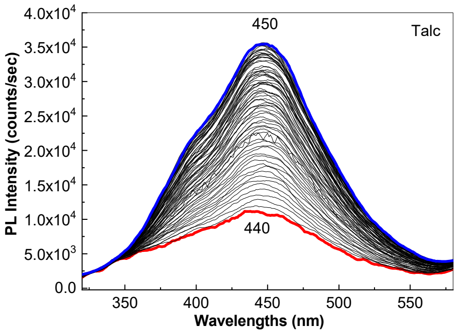

(**d1**)

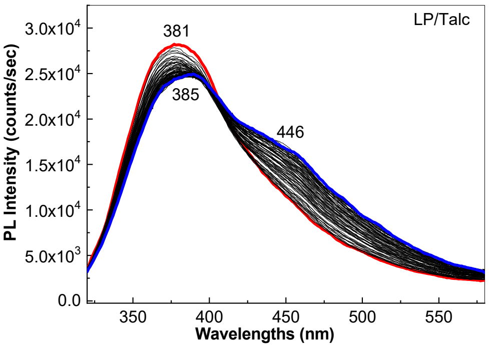

(**d2**)

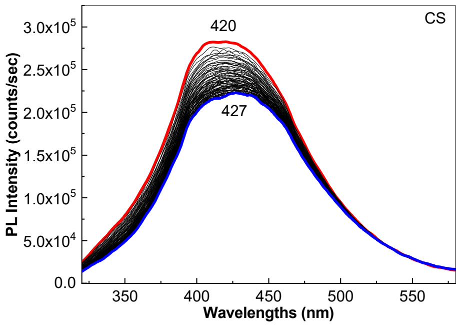

($\mathbf{e}_1$)

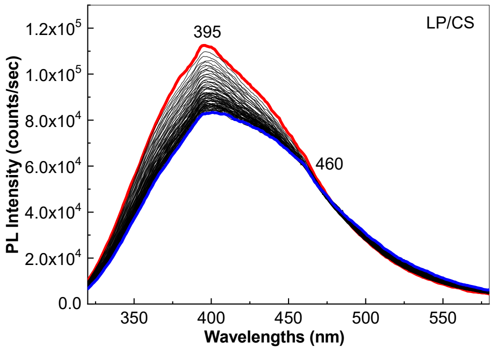

($\mathbf{e}_2$)

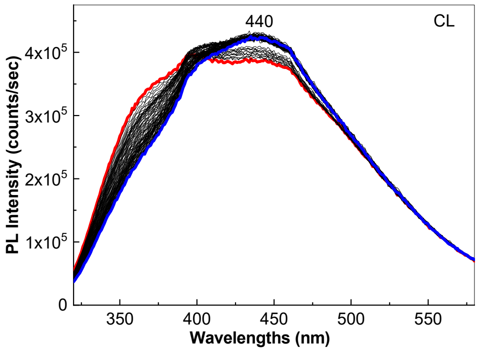

($\mathbf{f}_1$)

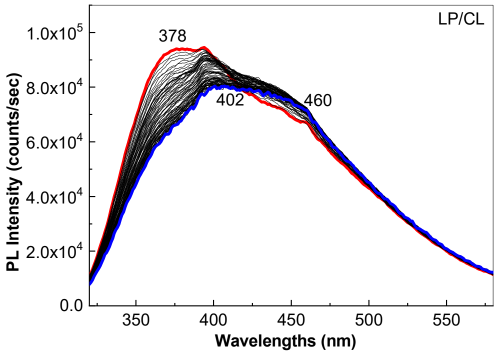

($\mathbf{f}_2$)

Figure S1. PL spectra of the LP (**a**), MS ($\mathbf{b}_1$), SiO_2 ($\mathbf{c}_1$), talc ($\mathbf{d}_1$), corn starch ($\mathbf{e}_1$) and cellulose ($\mathbf{f}_1$) and their blend with LP, i.e., LP/MS ($\mathbf{b}_2$), LP/SiO_2 ($\mathbf{c}_2$), LP/talc ($\mathbf{d}_2$), LP/corn starch ($\mathbf{e}_2$) and LP/cellulose ($\mathbf{f}_2$) as well as their evolution when the samples are exposed to UV light, for 187 min. All PL spectra are recorded at the excitation wavelength of 300 nm. The red, black and blue curves correspond to the PL spectra of samples before, in the intermediate state and after 187 min. of UV light exposure.
